# Level II (IIA/IIB) Lymph Node Evaluation in Head and Neck Cancer: A Retrospective Cohort Study from a Non-Endemic Region

**DOI:** 10.3390/jcm15031068

**Published:** 2026-01-29

**Authors:** Pınar Atabey, Ferit Aslan, Caner Kılıç, Özgen Yıldırım, Samet Özlügedik

**Affiliations:** 1 Department of Otorhinolaryngology, Head and Neck Surgery, Yüksek İhtisas University Affiliated Medicalpark Ankara Hospital, 06680 Ankara, Türkiye; pinaratabey16@gmail.com; 2 Department of Medical Oncology, Yüksek İhtisas University Affiliated Medicalpark Ankara Hospital, 06680 Ankara, Türkiye; 3 Department of Otorhinolaryngology, Ankara Dr. Abdurrahman Yurtaslan Oncology Training and Research Hospital, 06200 Ankara, Türkiye; canerkilic80@gmail.com; 4 Department of Medical Oncology, University of Health Sciences Ankara Etlik City Hospital, 06170 Ankara, Türkiye; ozgenayildirim@gmail.com; 5 Department of Otorhinolaryngology, Head and Neck Surgery, University of Health Sciences Ankara Etlik City Hospital, 06170 Ankara, Türkiye; sametozlugedik@yahoo.com

**Keywords:** head and neck squamous cell carcinoma, lymph node metastasis, level II lymph nodes, overall survival, disease-free survival, prognostic factor

## Abstract

**Background**: Head and neck squamous cell carcinoma (HNSCC) remains a major global health concern, with cervical lymph node metastasis being one of the most important determinants of prognosis. Level II (2A/2B) lymph nodes, in particular, play a key role in disease spread and survival outcomes. This study aimed to assess the prognostic impact of level II lymph node metastasis and evaluate the concordance between clinical and pathological staging in patients undergoing neck dissection. **Methods**: We retrospectively analyzed 138 non-metastatic HNSCC patients treated between 2007 and 2015. Clinical staging was standardized according to the AJCC 8th edition. Level II lymph nodes were dissected and evaluated separately by two independent pathologists. Survival outcomes were assessed using Kaplan–Meier analysis and Cox proportional hazards models. Correlation between clinical and pathological staging was examined using Pearson correlation analysis. **Results**: The median follow-up was 55.6 months. The 5-year overall survival (OS) and disease-free survival (DFS) rates were 62.4% and 60.1%, respectively. There was a strong correlation between clinical and pathological staging (r = 0.871, *p* < 0.001). Patients with level II metastasis had significantly worse outcomes, with median OS of 27 months versus an estimated 128 months among those without involvement (*p* = 0.008), and median DFS of 17.3 versus 114 months (*p* = 0.004). Age was identified as an independent predictor of mortality in multivariate analysis. **Conclusions**: Metastasis to level II lymph nodes is a strong adverse prognostic factor in HNSCC. These findings highlight the importance of detailed nodal evaluation in guiding neck dissection strategy, risk stratification, and subsequent adjuvant treatment decisions.

## 1. Introduction

Head and neck squamous cell carcinoma (HNSCC) represents a major global health challenge, accounting for 5–10% of all malignancies worldwide and exerting a substantial burden on morbidity and mortality [1]. Cervical lymph node metastasis remains the most powerful predictor of prognosis in HNSCC, influencing not only overall survival (OS) but also locoregional control, distant dissemination, and decisions regarding adjuvant therapy [2,3]. Even a single metastatic lymph node has been shown to reduce survival by nearly 50% [3], underscoring the critical need for accurate staging and nodal assessment. Over the past two decades, neck dissection has evolved from a radical approach to more selective procedures aimed at reducing morbidity while maintaining oncologic adequacy [4,5,6].

According to the Robbins classification adopted by the American Head and Neck Society and the American Academy of Otolaryngology–Head and Neck Surgery, the cervical lymphatic system is divided into five anatomical levels based on consistent fascial and muscular landmarks. Level I includes the submental and submandibular nodal basins; Level II comprises the upper jugular group; Level III the mid-jugular nodes; Level IV the lower jugular nodes; and Level V the posterior triangle nodes. This standardized classification provides a reproducible framework for evaluating metastatic patterns and planning surgical neck dissection [7].

Level II lymph nodes are anatomically divided into level IIA and IIB based on their relationship to the spinal accessory nerve (SAN). Level IIA nodes lie anterior and inferior to the SAN, whereas level IIB nodes are located posterior and superior to the nerve within the fatty compartment of the upper posterior triangle. This distinction is clinically important because the risk of metastasis differs between the two sublevels. While level IIA metastasis is relatively common in supraglottic, oropharyngeal, and oral cavity cancers, level IIB involvement is considerably less frequent, with reported rates typically ranging between 2 and 5% in large series. The anatomical position of level IIB behind the SAN also raises concerns regarding shoulder morbidity when dissected, making the decision to remove this compartment a matter of ongoing clinical debate. Among the cervical nodal basins, level II (particularly levels 2A and 2B) is a key site of metastasis for tumors of the supraglottic larynx, base of tongue, and oral cavity, and its involvement has been repeatedly associated with significantly poorer outcomes [8,9,10,11,12].

However, the optimal extent of elective neck dissection (END) in clinically node-negative (cN0) patients remains controversial. Several large studies have demonstrated survival benefits with END [13,14,15,16], yet concerns regarding shoulder dysfunction, cosmetic morbidity, and quality of life persist [5]. Consequently, a more personalized approach to nodal management has emerged, incorporating newly recognized prognostic indices such as the lymph node ratio (LNR) and the log odds of positive nodes (LODDS), which may refine risk stratification beyond traditional TNM classification [11,12,13]. In addition, contemporary real-world datasets, including multicenter experiences from non-endemic regions and immunotherapy-treated cohorts, have highlighted the importance of nodal research burden and anatomical distribution as key prognostic variables in head and neck cancers [16,17,18].

Recent literature further emphasizes the prognostic weight of nodal topography. A 2022 systematic review demonstrated that metastasis to level IIb is uncommon but consistently associated with inferior OS and DFS, supporting the selective preservation of level IIb only in carefully chosen cases [19]. Another 2023 multicenter analysis showed that the incorporation of LODDS or LNR significantly improves survival prediction compared with conventional nodal staging, especially in patients with limited nodal disease [20]. Moreover, emerging machine learning models developed between 2022 and 2024 have shown superior performance in predicting occult lymph node metastasis and identifying high-risk cervical levels compared with clinician-based assessment alone, suggesting that anatomical patterns of spread, including level II status, may soon be integrated into personalized surgical decision-making [21].

Given these evolving insights, a deeper understanding of the relationship between clinical and pathological staging and the prognostic consequences of level II (2A/2B) lymph node involvement is essential for optimizing neck dissection strategies. We hypothesized that (i) there is a strong concordance between clinical and pathological staging and (ii) metastasis to level II lymph nodes represents an independent adverse prognostic factor that substantially reduces OS and DFS in patients with HNSCC. This study aims to address these key questions using long-term survival data derived from a real-world cohort.

## 2. Materials and Methods

### 2.1. Study Design and Ethical Approval

This retrospective cohort study included patients who underwent neck dissection for head and neck squamous cell carcinoma (HNSCC) between January 2007 and December 2015. Follow-up data were reviewed through December 2022 to ensure adequate long-term survival assessment. Although retrospective in design, the methodological approach adhered to principles of prospective observational studies, with predefined variables, standardized data extraction, and independent validation of key outcomes. The study protocol was approved by the Regional Ethics Committee (Approval number: 369/2015; Approval date: 10 August 2015), and all procedures were carried out in accordance with the Declaration of Helsinki. Due to the retrospective nature of the analysis, the requirement for informed consent was waived.

### 2.2. Patient Selection

A total of 143 patients with histologically confirmed, non-metastatic (M0) primary HNSCC were included. Eligibility criteria were as follows: (i) age ≥ 18 years, (ii) no distant metastasis at diagnosis, (iii) availability of complete surgical and pathological records, and (iv) receipt of therapeutic or elective neck dissection. Exclusion criteria included: (i) recurrent tumors at presentation, (ii) prior neck surgery or radiotherapy to the cervical region, (iii) synchronous second primary tumors, and (iv) incomplete pathological evaluation of level II (2A/2B) lymph nodes. All stage IV patients included in this study were non-metastatic (M0). Stage IV classification reflected locally advanced primary tumors or nodal disease (T4 and/or N2–N3), as defined by the AJCC 8th edition. Preoperative evaluation consisted of physical examination, laryngoscopic assessment, and cross-sectional imaging (contrast-enhanced CT, MRI, and/or PET-CT). To ensure staging accuracy and comparability, all clinical staging data were reclassified according to the AJCC 8th edition [22].

The study included only histologically confirmed head and neck squamous cell carcinomas. Because HNSCC encompasses multiple anatomical subsites with inherently low case numbers in some categories, further subdivision into small anatomical groups would have produced statistically underpowered subgroups and deviated from the primary aim of evaluating level II metastatic behavior.

Recurrence data were collected in aggregate form, and detailed characterization of recurrence type (local, regional, distant) was not performed, as recurrence analysis was not a predefined study endpoint.

All tumors were restaged according to the 8th edition of the AJCC Cancer Staging Manual. Restaging was performed retrospectively using data extracted from operative notes, radiological reports, and pathology records. T category was reassigned based on maximum tumor diameter and, when available, depth of invasion (DOI); N category was reassigned using the number of metastatic lymph nodes, laterality, and the presence of extranodal extension (ENE). Only cases with complete data for tumor size, nodal status, and ENE were included in the final restaging analysis. M category was assigned according to clinical and radiological documentation at diagnosis. This process ensured a uniform and contemporary staging framework across the entire cohort.

### 2.3. Surgical Technique

Neck dissection procedures were performed by experienced head and neck surgeons following contemporary oncologic and anatomical standards. The extent of neck dissection—selective, modified radical, or radical—was determined based on clinical nodal status, primary tumor site, surgeon preference, and multidisciplinary tumor board recommendations. Resected lymph nodes were separated by laterality (right/left). Level II lymph nodes were further subdivided intraoperatively into level 2A and 2B by identifying the spinal accessory nerve (SAN) and the posterior boundary of the internal jugular vein. Each subgroup was placed in individually labeled containers to ensure precise pathological evaluation and prevent level misclassification. In cases of elective neck dissection (cN0), levels 1–3 or 1–4 were routinely included according to tumor location, while therapeutic dissections (cN+) incorporated additional levels as indicated. The extent of neck dissection was categorized using standard anatomical levels. Level 1–3 dissections involved removal of lymph node levels I, II, and III; level 1–4 included levels I–IV; level 2–4 consisted of selective removal of levels II–IV; and level 1–5 included levels I–V. These categories were used consistently throughout the study to analyze the prognostic impact of different surgical extents.

Although neck dissections were performed by more than one surgeon during the 2007–2015 period, all procedures were conducted within the same institution by surgeons trained in the same head and neck surgery program, resulting in a relatively homogeneous surgical approach and philosophy

### 2.4. Surgical and Oncologic Treatment Details

Treatment approaches reflected real-world practice during the study period (2007–2015). Surgical procedures were performed according to contemporary institutional standards, including selective, modified radical, or comprehensive neck dissection depending on tumor extent and surgeon preference. Radiotherapy was delivered using 3D conformal radiotherapy (3D-CRT) in the earlier years, with a transition to intensity-modulated radiotherapy (IMRT) after 2010. Systemic treatment, when indicated, consisted of platinum-based chemotherapy consistent with NCCN-aligned protocols used at the time. During the study period (2007–2015), elective neck dissection (END) was routinely performed for clinically N0 tumors arising from regions with a known risk of occult metastasis, in accordance with contemporary national practice patterns and institutional protocols.

### 2.5. Histopathological Evaluation

All specimens were evaluated independently by two board-certified head and neck pathologists. All retrieved lymph nodes were entirely submitted for histopathologic examination. Nodes were serially sectioned at 2–3 mm intervals perpendicular to the long axis, routinely processed, and examined on hematoxylin- and eosin-stained slides. At least one slide per tissue block was reviewed, and any metastatic focus—regardless of size—was considered positive; micrometastases (<2 mm) and isolated tumor cells (ITCs) were recorded when present. Level II positivity was defined as metastasis in either sublevel IIA or IIB and required concordance between both pathologists; discordant assessments were resolved via multi-headed microscopic review. Additional pathological parameters, including extranodal extension (ENE), the number of positive lymph nodes, total lymph nodes harvested per neck level, lymph node ratio (LNR), and the largest metastatic focus size, were documented, although these variables were not primary study endpoints.

### 2.6. Clinical Variables and Follow-Up

Demographic and clinical data were extracted from electronic hospital archives and operative reports. Variables included age, sex, smoking history, tumor site, TNM stage, type of neck dissection, pathological nodal status, receipt of adjuvant therapy, recurrence, and survival outcomes. Overall survival (OS) was defined as the time from diagnosis to death from any cause. Disease-free survival (DFS) was defined as the interval between diagnosis and first recurrence or death. All recurrence events were confirmed radiologically and/or histopathologically.

### 2.7. Statistical Analysis

Statistical analyses were conducted using IBM SPSS Statistics version 22.0 (IBM Corp., Armonk, NY, USA). Continuous variables were tested for normality using the Kolmogorov–Smirnov test. Descriptive statistics were expressed as mean ± standard deviation or median (range), as appropriate. Categorical variables were summarized using frequencies and percentages. In subgroups where the Kaplan–Meier curves did not reach the 50% survival probability due to a high proportion of censored observations, median survival could not be estimated; therefore, mean survival times with 95% confidence intervals were reported. Comparisons of survival outcomes were performed using Kaplan–Meier analysis, with differences assessed by using the log-rank test. Because the number of survival events was insufficient to support a stable multivariable model, Cox proportional hazards regression was limited to exploratory univariate analyses to evaluate crude associations only. Multivariable Cox regression was not performed to avoid generating unstable or non-interpretable estimates. A correlation analysis between clinical and pathological staging was performed using Pearson’s correlation coefficient. A *p*-value < 0.05 was considered statistically significant.

### 2.8. Use of Generative AI

During the preparation of this manuscript, a large language model-based tool (ChatGPT 5.1, OpenAI) was utilized to assist with language refinement, grammar correction, and overall text organization. No AI-generated data, statistical analysis, scientific interpretation, or original results were produced, and all scientific content, methodology, and conclusions were developed solely by the authors.

## 3. Results

A total of 138 patients with primary head and neck squamous cell carcinoma (HNSCC) who underwent neck dissection were included in the study. The median age was 57 years (range, 30–81), and the majority were male (85.5%, *n* = 118). A smoking history was present in 92% of cases (*n* = 127) (Table 1). HPV/p16 immunohistochemistry data were unavailable for this cohort.

Regarding tumor localization, the most common site was the supraglottic larynx (56.5%, *n* = 78), followed by the tongue (12.5%, *n* = 17), lip (10.9%, *n* = 15), and glottic larynx (8.7%, *n* = 12). Pathological staging revealed 3.6% (*n* = 5) stage I, 41.3% (*n* = 57) stage II, 42.9% (*n* = 69) stage III, and 10.1% (*n* = 14) stage IV disease. T4 tumors were identified in 8.7% (*n* = 12), and lymph node positivity was observed in 14.5% (*n* = 20) (Table 1).

Of the 138 patients, 102 (71.3%) presented as clinically N0 based on at least two imaging modalities (CT, MRI, and/or PET-CT). Among these clinically N0 cases, only 1 patient (0.9%) was found to have pathological nodal metastasis (cN0 → pN+), indicating a low rate of occult metastasis. Conversely, 21 clinically N+ patients (20.6%) were found to be pathologically node-negative (pN0) after neck dissection. Stage I (T1N0M0) and Stage II (T2N0M0) case counts are provided in Table 1. These findings highlight the discordance between clinical and pathological nodal assessment in this cohort.

Among the 20 patients with pathological nodal metastasis, the majority (81.0%) involved level II. The remaining metastases were distributed across other cervical levels in very small numbers that did not permit meaningful subgroup analysis.

With respect to surgical management, 35.5% (*n* = 49) underwent ipsilateral neck dissection, while 64.5% (*n* = 89) underwent bilateral dissection. The most common extent of dissection was level 2–4 (62.3%, *n* = 86), followed by level 1–4 (19.6%, *n* = 27), level 1–3 (10.1%, *n* = 14), and level 1–5 (8%, *n* = 11). Postoperatively, 27.5% (*n* = 38) received adjuvant chemotherapy and 17% (*n* = 18) received adjuvant chemoradiotherapy (Table 2).

For clarity, we reiterate that the median follow-up duration of the study population was 55.6 months (range, 0.72–157 months), and this time frame was used consistently for all OS and DFS calculations.

**Figure 1:**
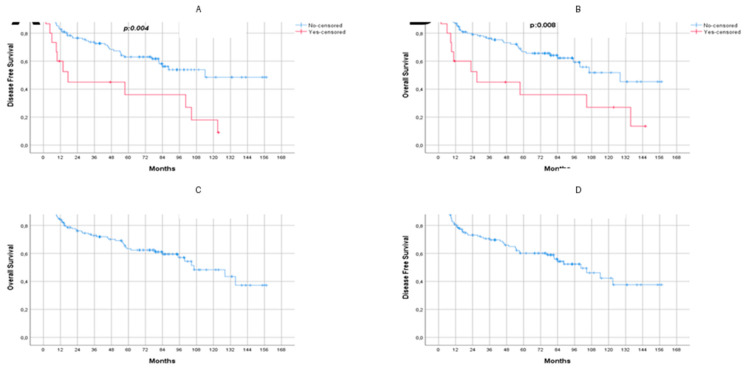
Kaplan–Meier survival analyses in patients with HNSCC. (**A**) Disease-free survival (DFS) by level II (2A/2B) lymph node involvement. (**B**) Overall survival (OS) by level II involvement. (**C**) Overall OS in the entire cohort. (**D**) Overall DFS in the entire cohort. Colored curves represent survival probabilities over time; solid lines indicate level II-negative patients and dashed lines indicate level II-positive patients. All panels have been enlarged and reformatted for clarity.

The median follow-up duration was 55.6 months (range, 0.72–157 months). During follow-up, 37.7% (*n* = 52) of patients died, and 40.6% (*n* = 56) developed recurrence. In the overall cohort, the 5- and 10-year overall survival (OS) rates were 62.4% (SE: 0.44) and 43.5% (SE: 0.73), respectively, with median OS of 106.3 months (95% CI: 76.1–136.5). The 5- and 10-year disease-free survival (DFS) rates were 60.1% (SE: 0.45) and 37.6% (SE: 0.74), with median DFS of 100.4 months (95% CI: 74.2–126.5) (Figure 1).

Cox regression analysis revealed a significant association between age and mortality (*p* = 0.002; HR: 1.044; 95% CI: 1.016–1.072). A strong positive correlation was observed between clinical and pathological staging (Pearson’s r = 0.871, *p* < 0.001).

Survival analyses according to the extent of neck dissection (levels 1–3, 1–4, 2–4, and 1–5) demonstrated statistically significant differences in both disease-free survival (DFS) and overall survival (OS). DFS was longest in patients who underwent level 2–4 dissection (mean 100.7 months; 95% CI: 85.4–115.8) and shortest in the level 1–5 group (mean 35.7 months; 95% CI: 18.0–53.3), with a significant difference among groups (log-rank *p* = 0.009). Similarly, OS differed significantly (log-rank *p* = 0.010), with level 2–4 and level 1–3 dissections showing the most favorable outcomes (mean OS 101.1 and 102.8 months, respectively), while level 1–5 dissections had the poorest survival (mean OS 45.5 months; 95% CI: 25.6–65.4). These findings indicate that the extent of neck dissection is associated with long-term oncologic outcomes in this cohort.

Survival analysis according to level II lymph node involvement showed markedly inferior outcomes in patients with metastasis. Median OS was 27 months (95% CI: 0–55.2) in patients with level II involvement compared to an estimated 128 months in those without (*p* = 0.008). Similarly, median DFS was 17.3 months (95% CI: 4.1–30.0) versus 114 months (*p* = 0.004). N+ status was not independently associated with OS/DFS in univariate analysis.

## 4. Discussion

In this retrospective cohort of patients with head and neck squamous cell carcinoma (HNSCC), we demonstrated a strong correlation between clinical and pathological staging, supporting the reliability of clinical evaluation in guiding surgical decisions. More importantly, we identified level II (2A/2B) lymph node involvement as an independent adverse prognostic factor significantly reducing both overall survival (OS) and disease-free survival (DFS). These findings are consistent with previous reports showing that even a single positive lymph node can decrease survival by nearly 50% [3].

The role of elective neck dissection (END), particularly in clinically node-negative (cN0) patients, remains controversial. López et al. highlighted the efficacy of selective neck dissection in clinically node-positive patients [2], while Xu et al. reported survival benefits of END in cN0 oral squamous cell carcinoma, particularly among older patients [5]. Large series have also confirmed the survival advantage of END for OS and disease-specific survival [13,14,15]. The strong correlation observed between clinical and pathological staging in our cohort further supports the appropriateness of surgical intervention in selected patients.

Although elective neck dissection (END) has historically been used in clinically N0 head and neck cancer, it increasingly raises concern for overtreatment. A substantial proportion of ENDs yield pathologically negative lymph nodes, exposing patients to avoidable functional and esthetic morbidity such as shoulder dysfunction without clear survival benefit in low-risk cases [13]. In our cohort, 85.5% of patients were pN0, emphasizing the importance of refining patient selection and adopting more risk-adapted approaches to minimize unnecessary surgery.

In our cohort, the rate of occult metastasis among clinically N0 patients was extremely low (0.9%). Although this is lower than most reported series, similar findings have been described, with some early-stage HNSCC cohorts demonstrating occult metastasis rates below 5% [23,24]. Conversely, 20 clinically N+ cases were confirmed to be pathologically node-negative, which may reflect false-positive imaging findings due to inflammatory or reactive lymphadenopathy, a limitation well described in PET/CT-based nodal assessment [25]. These discrepancies highlight the inherent limitations of radiologic nodal staging and may partly explain the high proportion of patients who underwent elective neck dissection despite ultimately being pN0. Such findings reinforce the importance of individualized risk stratification in early-stage disease.

In the present study, both DFS and OS varied significantly according to the extent of neck dissection. Patients who underwent level 2–4 dissections experienced the most favorable survival, whereas those treated with level 1–5 dissections had the poorest outcomes. Although this pattern may partly reflect selection bias where wider dissections are typically performed in patients with more aggressive or extensive disease, it also suggests that surgical strategy and nodal basin involvement have prognostic relevance beyond level II status alone. The finding aligns with emerging evidence indicating that the anatomical distribution and extent of nodal clearance can influence recurrence patterns and long-term survival. Nonetheless, given the retrospective nature of our study, these results should be interpreted cautiously and validated in larger multicenter cohorts.

The prognostic significance of level II involvement represents the most striking finding of our study. Patients with metastasis to level II nodes had a median OS of only 27 months and DFS of 17.3 months, compared to significantly longer outcomes in those without such involvement. This observation aligns with prior studies showing that metastasis to level II, particularly level IIb, is relatively uncommon but strongly associated with poor prognosis [6,8]. Mamelle et al. also emphasized that not only the presence but also the anatomical location of nodal metastasis should be considered as a vital prognostic parameter [16]. Our findings highlight that anatomical patterns of nodal involvement significantly influence survival outcomes in HNSCC. Similar observations regarding disease behavior and treatment response in head and neck malignancies have been demonstrated in regional multicenter cohorts, particularly in nasopharyngeal carcinoma from non-endemic regions [17], underscoring the importance of population-based real-world evidence. Our data reinforce the importance of incorporating the anatomical distribution of nodal disease into treatment planning.

Recent evidence further supports the prognostic importance of anatomical nodal distribution in optimizing surgical strategies. In a 2024 study published in *J. Clin. Med.*, Sung et al. demonstrated that tailoring the extent of neck dissection based on the precise anatomical mapping of metastatic cervical lymph nodes—particularly those involving level II—improves oncologic decision-making while minimizing unnecessary surgical morbidity [26,27]. Their findings align with our results and reinforce that nodal topography, not merely nodal positivity, should be integrated into individualized surgical planning for patients with HNSCC.

Emerging nodal parameters such as lymph node ratio (LNR) and log odds of positive lymph nodes (LODDS) have recently gained attention for their prognostic value in head and neck cancers [28]. Although these indices were not directly evaluated in our study, the pronounced negative impact of level II involvement underscores the clinical importance of nodal burden and distribution, suggesting that integration of such metrics into future analyses could enhance prognostic stratification. Moreover, real-world multi-center data evaluating immunotherapy in recurrent or metastatic head and neck cancer have similarly highlighted the prognostic relevance of disease burden and treatment selection in this population [18]. Future prospective studies integrating LNR/LODDS and machine learning-based nodal prediction models may refine risk stratification beyond anatomical descriptors.

In addition to traditional pathological parameters, recent AI-assisted radiomics studies have advanced our understanding of nodal behavior in head and neck cancers. Chen et al. demonstrated that habitat radiomics models can accurately predict occult lymph node metastasis and simultaneously characterize immune microenvironmental features associated with nodal spread, suggesting a biological continuum between tumor phenotype and metastatic pattern [29]. Supporting these findings, a 2025 systematic review and meta-analysis by Valizadeh et al. confirmed that radiomics and artificial intelligence models exhibit high diagnostic accuracy for detecting cervical nodal metastasis in HNSCC, highlighting their potential role in refining surgical indications, including the selection of patients for elective versus extended neck dissection [30]. These developments suggest that future treatment algorithms may increasingly integrate radiomics-based nodal risk assessment with anatomical mapping such as level II status, thereby improving precision in both staging and therapeutic planning.

Several studies have attempted to explain why level II metastasis is associated with particularly poor outcomes. Level II nodes receive lymphatic drainage from the base of tongue, supraglottis, and oropharynx—sites known for aggressive tumor biology and early extracapsular extension. Moreover, emerging radiomics and molecular profiling data suggest that level II nodes may harbor immune-cold microenvironments characterized by stromal fibrosis, reduced T-cell infiltration, and early nodal remodeling. These biological characteristics may partly explain the strong negative prognostic impact observed in patients with level II involvement [31].

Additionally, age was identified as an independent predictor of mortality, in line with prior evidence indicating that older patients often face greater risks related to both surgery and adjuvant treatment. This highlights the need for multidisciplinary tumor board discussions when making therapeutic decisions in elderly populations.

The strengths of our study include a relatively long median follow-up duration (55.6 months) and histopathological assessment performed by two independent pathologists. However, the study is limited by its retrospective design, single-center setting, and relatively small sample size, which may restrict the generalizability of the findings.

In summary, our study confirms the strong concordance between clinical and pathological staging and demonstrates that level II lymph node involvement is a powerful independent predictor of poor OS and DFS in patients with HNSCC. These findings highlight the need to consider both the presence and anatomical distribution of nodal metastasis when tailoring neck dissection strategies and planning adjuvant therapy.

## 5. Study Limitations and Future Perspectives

This study has several limitations. Its retrospective single-center design may limit generalizability, and the relatively small number of patients with level II metastasis reduces the statistical power of subgroup analyses. Potential confounders including comorbidities, HPV/p16 status, and heterogeneity in adjuvant treatment could not be fully controlled. In addition, the study period (2007–2015) coincided with major transitions in surgical techniques, radiotherapy modalities, and systemic therapies, which may have influenced survival outcomes.

Future studies should validate these findings in larger multicenter cohorts and incorporate quantitative nodal indices such as lymph node ratio (LNR) and log odds of positive lymph nodes (LODDS). Emerging radiomics and artificial intelligence-based approaches may further enhance risk stratification by integrating anatomical, pathological, and biological nodal characteristics, ultimately enabling more personalized neck dissection strategies in HNSCC.

## 6. Conclusions

This study demonstrates a strong concordance between clinical and pathological staging in patients with head and neck squamous cell carcinoma, supporting the reliability of clinical assessment in guiding the extent of neck dissection. Importantly, metastasis to level II lymph nodes—particularly levels IIA and IIB—was identified as a powerful adverse prognostic factor, significantly reducing both overall survival and disease-free survival. These findings highlight that anatomical nodal distribution, rather than nodal positivity alone, should play a central role in surgical planning and decisions regarding adjuvant therapy. Incorporating level II status into routine risk stratification may help optimize neck dissection strategies and improve long-term oncologic outcomes.

## Figures and Tables

**Table 1:** Baseline Demographic and Clinical Characteristics of Patients.

Characteristic	*n* (%)
**Age, years (range)**	57 (30–81)
**Sex**	
Female	20 (14.5)
Male	118 (85.5)
**Smoking status**	
Smoker	127 (92)
Non-Smoker	11 (8)
**Primary tumor site**	
Supraglottic larynx	78 (56.5)
Glottic larynx	12 (8.7)
Lip	15 (10.9)
Tongue	17 (12.3)
Floor of mouth	7 (5.1)
Parotid gland	6 (4.3)
Maxilla	3 (2.2)
**Lymph node metastasis (N1–N2)**	
Present	20 (14.5)
Absent	118 (85.5)
**T4 tumors**	
Present	12 (8.7)
Absent	126 (91.3)
**Stage (AJCC 8th)**	
Stage 1	5 (3.6)
Stage 2	57 (41.3)
Stage 3	62 (42.9)
Stage 4a	14 (10.1)

**Table 2:** Surgical features, pathological findings, adjuvant treatments, and follow-up outcomes.

Characteristic	*n* (%)
**Type of neck dissection**	
Ipsilateral	49 (35.5)
Bilateral	89 (64.5)
**Extent of lymph node dissection**	
Level 2–4	86 (62.3)
Level 1–4	27 (19.6)
Level 1–3	14 (10.1)
Level 1–5	11 (8)
**Level IIa lymph nodes**	
Positive	12 (8.7)
Negative	126 (91.3)
**Level IIb lymph nodes**	
Positive	4 (2.9)
Negative	134 (97.1)
**Adjuvant therapy**	
Present	43 (31.2)
Absent	95 (68.8)
**Adjuvant chemotherapy**	
Present	38 (27.5)
Absent	100 (72.5)
**Chemoradiotherapy (CRT)**	
Present	18 (17)
Absent	120 (83)
**Recurrence**	
Present	56 (40.6)
Absent	82 (59.4)
**Mortality**	
Yes	52 (37.7)
No	86 (62.3)
**Follow-up, months (range)**	55.6 (0.72–157)

## Data Availability

The datasets generated and/or analyzed during the current study are available from the corresponding author upon reasonable request.
